# Modeling the Transitional Phase of Epithelial Cells Reveals Prognostic and Therapeutic Targets in Pancreatic Ductal Adenocarcinoma

**DOI:** 10.3390/cancers17111813

**Published:** 2025-05-29

**Authors:** Linhan Ye, Zongyao Chen, Jingcheng Zhang, Qiaolin Li

**Affiliations:** 1 Department of Surgery, Klinikum rechts der Isar, School of Medicine, Technical University of Munich, 81675 Munich, Germany; linhan.ye@tum.de (L.Y.); zongyao.chen@tum.de (Z.C.); jingcheng.zhang@tum.de (J.Z.); 2 Department of Hematology, Oncology and Tumor Immunology, Charité University Medicine Berlin, Campus Virchow Clinic, 13353 Berlin, Germany

**Keywords:** pancreatic ductal adenocarcinoma progression, transitional epithelial cells, risk stratification model, tumor microenvironment remodeling, immune infiltration

## Abstract

**Background:** Pancreatic ductal adenocarcinoma (PDAC) is a highly aggressive cancer with poor prognosis, and its progression is driven by epithelial plasticity and tumor microenvironment remodeling. Finding biomarkers that are responsible for the turning point from the early stage to the aggressive phase would facilitate clinical management. **Method:** In this study, we employed single-cell RNA sequencing to characterize a distinct subpopulation of highly proliferative epithelial cells undergoing a transitional phase during PDAC progression. By linking to cell cycle dysregulation, epithelial differentiation, and clinical staging, we constructed a gene-based risk score model using Lasso Cox regression. The expression of selected genes within the model was further validated using qPCR. **Results:** The model demonstrated robust predictive power for patient prognosis, TNM staging, and chemotherapy sensitivity. Further analysis of the tumor microenvironment revealed intensified crosstalk between a specific fibroblast subpopulation and transitional epithelial cells, mediated largely by collagen signaling. This stromal–epithelial interaction was found to contribute to the fibrotic barrier characteristic of PDAC. Additionally, immune profiling uncovered altered infiltration patterns, particularly involving natural killer (NK) cells in high-risk patients, suggesting mechanisms of immune tolerance and inhibition. **Conclusions:** These findings offer potential avenues for early detection, risk stratification, and targeted therapeutic strategies in PDAC.

## Simple Summary

Pancreatic cancer is a highly aggressive disease that is often diagnosed too late for effective treatment. This study focuses on a group of epithelial cells that appear to undergo a key transition during early tumor development. By closely examining these cells, we identified specific gene patterns that can be used to assess disease severity and predict patient outcomes. The findings also highlight how certain fibroblasts in the tumor environment may contribute to cancer progression by influencing these transitional cells. Moreover, differences in immune cell presence, especially natural killer cells, were observed in patients with higher risk levels. Together, these insights may support earlier diagnosis, more accurate risk assessment, and the development of tailored treatment approaches for pancreatic cancer.

## 1. Introduction

Pancreatic cancer (PCa) is one of the most aggressive cancers, characterized by extremely high mortality rates with a dismal 5-year survival rate of around 12% [1]. As the predominant form of pancreatic neoplasms, PDAC accounts for more than 90% of all diagnosed PCa cases [2]. A major challenge in treating PDAC is the lack of early diagnosis and the limited efficacy of current therapeutic strategies [3]. Only 20% of patients with stage I–II PCa are diagnosed at an earlier, resectable stage, while a majority of cases are identified at advanced stages (III–IV), where therapeutic options are significantly constrained [4]. Currently, surgery remains the curative treatment option for PDAC, but it is only applicable to patients diagnosed at the I–II stages.

Early-stage PDACs are often asymptomatic or present with nonspecific symptoms, making timely diagnosis particularly difficult in clinical practice [5]. In response, substantial efforts have been directed toward improving early detection through advances in imaging techniques, molecular biomarker identification, and integrated diagnostic strategies [6,7]. Despite these advances, the lack of effective early diagnostic tools remains a critical barrier to timely intervention and improved clinical outcomes.

During PDAC progression, tumor cells undergo increasing degrees of dedifferentiation and epithelial plasticity, which are often accompanied by enhanced proliferative activity and cell cycle deregulation [8,9,10]. Cell cycle dysregulation has been identified as a key driver of PDAC, characterized by uncontrolled cell cycle progression that promotes tumor proliferation and disease progression [9,11]. Notably, dysregulation of the G2/M checkpoint has been linked to advanced-stage PDAC and poor patient survival [12]. Therefore, elucidating the mechanisms underlying cell cycle dysregulation and associated gene networks represents a promising approach to improving PDAC diagnosis and early detection.

A dense, fibrotic stromal microenvironment represents one of the most prominent pathological features of PDAC [13,14]. The intercommunication between stromal compartment and tumor (epithelial) cells drives the development of PDAC [15]. Fibroblasts serve as the primary cellular elements in the stromal compartment, acting as the main producers and regulators of the ECM and collagen, driving its remodeling and significantly contributing to PDAC progression [16,17]. ECM remodeling forms the dense fibrotic stroma, which acts as a physical barrier that impedes the penetration of chemotherapy drugs and hinders immune cell infiltration [18]. Therefore, deciphering the interactions between the stroma and tumor is crucial for understanding the pathogenesis of PDAC and identifying potential therapeutic strategies to overcome these challenges.

The evolving immune landscape significantly supports both the initiation and progression of PDAC [19,20]. NK cells, as key components of the innate immune system, play a critical role in shaping the immune landscape of PDAC [21]. Studies have demonstrated a significant reduction in NK cell infiltration in human PDAC, with lower NK cell abundance correlating with poorer patient survival [22,23]. NK cells exist in resting and activated states. Activated NK cells are cytotoxic, while resting cells require stimulation to exert similar effects [24]. These differences mainly arise from variations in surface receptor expression. Spatial transcriptomic analyses further reveal that NK cells localize within the PDAC tumor microenvironment and interact closely with malignant epithelial ductal cells [25]. Collectively, the dynamic presence and functional heterogeneity of NK cells underscore their importance in PDAC progression and support their potential utility as prognostic biomarkers and immunotherapeutic targets.

In this study, single-cell RNA sequencing and bulk RNA sequencing were employed to delineate a subpopulation of highly proliferative epithelial cells that are in a transitional phase. By integrating the dysregulated cell cycle status and degree of cellular differentiation with the clinical stage, a gene-based risk score model was developed, which demonstrates the ability to predict both prognosis and clinical stage. Furthermore, utilizing this risk score, we conducted a comprehensive analysis of the cellular microenvironment, identifying augmented intercellular communication between a specific fibroblast subpopulation and epithelial cells, which was found to drive ECM remodeling, thereby revealing potential mechanisms underlying the progression of PDAC. Additionally, the performance of the risk score model was evaluated and validated in the context of various clinical outcomes, TNM staging, and chemotherapy response, offering significant predictive value for clinical treatment strategies. Finally, the correlation between immune cell infiltration patterns and the risk score model was investigated, specifically on the different infiltration patterns of NK cells between the high and low-risk group, offering insights into the mechanisms by which immune cells may contribute to tumor immune evasion.

## 2. Materials and Methods

### 2.1. Single Cell Analysis

Eleven single cell samples (4 in PDAC stage I [PDACI], 4 in stage II [PDAC II], and 3 in stage III [PDAC III]) from CAR001160 were obtained from the TISCH platform (http://tisch.comp-genomics.org) (accessed on 20 February 2023). Each sample was curated by performing scDblFinder to exclude the doublets, per-CellQCFilters to trim out the outliers (low quality cells) followed by the use of MAD (Median Absolute Deviation, MAD = 5) thresholds taking all the cells below median plus 5 times the MAD. Seurat objects were merged together for normalization in ‘LogNormalize’ method with a 10,000 scale. The maxLikGlobalDimEst function was applied to determine the appropriate dimensionality. Samples under different layers in Seurat V5 were integrated by HarmonyIntegration for batch correction. The FindMarkers function combined with the SingleR package was implemented to annotate the clusters.

### 2.2. Differentiation Analysis

Differentiation status of each single cell was evaluated by CytoTRACE2 (version 1.0.0) [26]. To include a sufficient number of single cells to be representative and statistically robust, cells with a relative differentiation score above 60% and below 40% were categorized into less differentiated group and more differentiated group, respectively. The rest were regarded as middle group.

### 2.3. Differential Expression Analysis

Differentially expression analysis was calculated by the limma-voom framework (version 3.62.1) [27] following a pseudobulk transformation. Only genes were included in the analysis if their expression counts exceeded 10 in at least three samples. Differentially expressed genes were defined based on the criteria of adjusted *p*-value (*p.adj*) < 0.05 and absolute log2 fold change > 1.

### 2.4. Continuous Expressed Gene Pattern Analysis Across PDAC Stages

TDEseq (version 1.1) [28] was employed to identify genes with temporal dynamic expression patterns in our PDAC stage-series single-cell RNA sequencing transcriptomic data. Counts, normalized counts, and metadata were extracted from a subset of Seurat objects to create a TDEseq object. Linear models were used to fit the expression data. A minimum fold change of 0.1 and a significance threshold of 0.05 were set for defining differential expression. The model type FastLMM was selected to account for possible confounding effects. Genes that exhibited a continuous increase in expression throughout the PDAC stages were labeled with the “SignificantDE” tag and classified under the “growth” pattern in the TDEseq output.

### 2.5. Functional Enrichment Analysis

Single-cell gene set enrichment analysis was conducted by pseudobulk transforming grouped by sample source and annotation, followed by the singleseqgset package. Single-sample gene set enrichment analysis was applied on ranked genes for each clinical sample. All the enrichment scores were shown in z-scored format. GO analysis was carried out using the clusterProfiler (version 4.14.4) [29] package for over-representative analysis.

### 2.6. Lasso Cox Regression Model Construction and Validation

The gene expression profiles and clinical data of GSE79668 were downloaded from the GEO database (https://www.ncbi.nlm.nih.gov/geo/) (accessed on 1 September 2024) as the test cohort, and PDAC TCGA PanCancer Atlas and PDAC QCMG data were download from the cBioPortal platform (https://www.cbioportal.org/) (accessed on 8 September 2021) as the validation cohort. To avoid the error of negative value in log2 transformation, transcriptome data in a z-score scale were used for the downstream analysis. To narrow down the number of input genes for the Lasso Cox model due to the overfitting issue, overlapped genes were fed to univariate Cox proportional hazards regression models first to test the survival correlation. Then, Lasso Cox regression was performed with 10-fold cross-validation to determine the optimal lambda. For the assessment of the accuracy and reliability of the survival model, Kaplan–Meier survival analysis and a further time-dependent receiver operating characteristic (ROC) analysis was utilized on validation cohort, and we chose the first 3 years as representative follow-up points for ROC analysis.

### 2.7. Evaluation of Association Between Risk Score and Clinical Outcomes

To investigate the association between the risk score and clinical outcomes such as overall survival, DFS (disease-free survival), PFS (progression-free survival), and DSS (disease-specific survival), a univariate Cox proportional hazards regression model was implemented using the coxph function from the survival package. Kaplan–Meier survival curves were then generated for each survival outcome based on the risk score-stratified groups to further illustrate the impact of our predictive model on survival. Log-rank tests were used to assess the statistical significance of survival differences between groups. Kruskal–Wallis tests were carried out for statistical analysis in the distribution of risk scores across clinical outcomes. Dunn’s post hoc tests were applied to perform pairwise comparisons. The results were adjusted for multiple comparisons using the Benjamini–Hochberg test.

### 2.8. Cell–Cell Communication Analysis

To investigate the mechanisms underlying the nature of epithelial cells categorized by risk score, we added a risk score category label to the epithelial cells and incorporated this information into the Seurat object. We then fed the entire Seurat object into CellChat (version 2.1.2) [30] to explore the interactions between the epithelial cell groups and other cell types in the dataset.

### 2.9. Immune Cell Infiltration Analysis

CIBERSORT was conducted for characterizing the immunol cellular composition from TCGA gene expression profiles using LM22 as a reference, which contained 547 gene signatures distinguishing 22 human immune cell phenotypes. Patient data were analyzed and relate to the risk score category, which infer the distribution of immune-infiltrating cells and its potential role in the diverse risk group.

### 2.10. Quantitative Polymerase Chain Reaction (Qpcr)

qPCR was performed using KAPA SYBR^®^ FAST qPCR kits. Each experiment was independently repeated three times, with GAPDH used as the internal control. The relative expression levels were calculated using the 2 − ΔΔCt method based on the CT values. The primer sequences were as follows: RPL39L forward: GAG-TGTTCCAATCTCTCCCTCA, RPL39L reverse: CCACAATACCCTCCTGATTACCC; C16orf74 forward: TCCTGAACGACAAGCACCTGGA, C16orf74 reverse: GGGTCGAT-TTCTCCATCATCTGG; UBE2C forward: CTGGCGATAAAGGGATTTCTGCC, UBE2C reverse: GCGAGAGCTTATACCTCAGGTC; GAPDH forward: GTCTCCTCTGACTTCAACAGCG, GAPDH reverse: ACCACCCTGTTGCTG-TAGCCAA.

## 3. Results

### 3.1. Identification of a Highly Proliferative Epithelial Subpopulation Driving Early Tumorigenesis in PDAC

**Figure 1:**
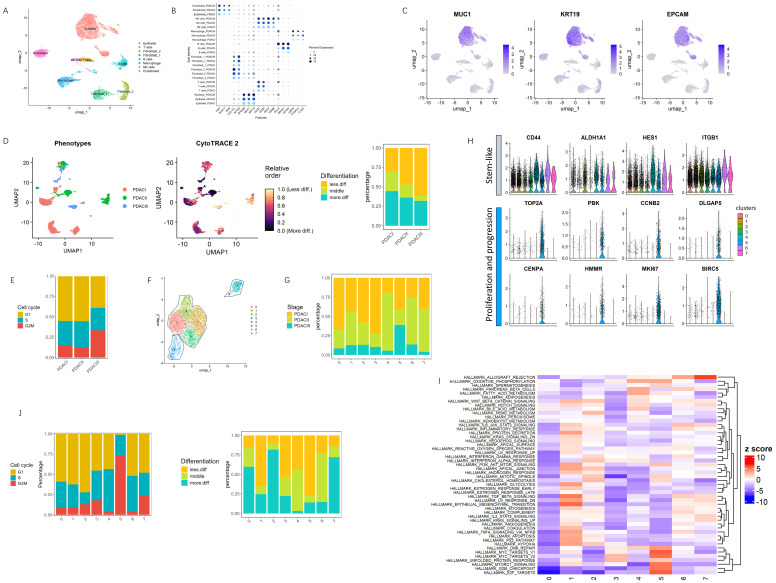
Overview of whole integrated single cell data and on re-analyzed epithelial cells. (**A**) Integrated PDAC single cell data in UMAP with annotation. (**B**) Dot plot representation of the expression of marker genes for annotation. The dot size represents the percentage of cells expressing each marker gene, and the dot color indicates PDAC stage: light blue for PDAC I, sky blue for PDAC II, and dark blue for PDAC III. (**C**) UMAP plots showing the expression level of epithelial cells marker genes. (**D**) UMAP analysis of epithelial cell differentiation and clinical stage in PDAC: UMAP embedding of epithelial cells grouped by clinical stage (left) and by corresponding CytoTRACE2 relative differentiation score (middle). The relative differentiation score ranges from 0 to 1. The higher the score, the lower the degree of differentiation and the brighter the corresponding cell color in UMAP. The right plot represents the distribution of differentiation group across PDAC stages. (**E**) Cell cycle dynamics in different PDAC stages. (**F**) Re-normalized and re-integrated epithelial cells in eight major clusters shown in UMAP. (**G**) Composition of PDAC grades across sub-clusters. (**H**) Expression of stem-like genes as well as proliferation and cancer progression-associated genes in epithelial sub-clusters. The X-axis represents clusters, and the Y-axis shows average expression levels. (**I**) GSEA on epithelial sub-clusters with Hallmark collection. (**J**) Distribution of cell cycling (left) and differentiation status (right) grouped by sub-clusters.

To explore the interesting genes driving the early tumorigenesis of PDAC disease, we analyzed 11 single-cell samples from the CAR001160 dataset, which represents different clinical stages. After quality control and doublets cleaning, the transcriptomes of 19,362 cells (7805 cells from PDACI, 7703 cells from PDAC II, and 3854 from PDAC III) were retained for subsequent analysis. Ultimately, analysis returned the identification of eight major cell populations based on the marker genes (Figure 1A,B).

Epithelial cells, as a key population, are widely considered as the primary cause of PDAC initiation and progression. A focused analysis on epithelial cells may provide more elaborate and more subtle biomarkers for the prognosis of PDAC. To this end, the analysis proceeded by extracting the epithelial cells as a key population using the three most well-known epithelial marker genes (Figure 1C), which were then re-normalized to ensure consistency across the dataset, and the differentiation status of single cells was assessed individually. It revealed that more advanced stages contained higher percentage of less differentiated cells as well as cells in the G2M phase (Figure 1D,E), indicating potential alterations in proliferative dynamics and cell cycle regulation. To further delineate the heterogeneity within the epithelial cell population, we re-integrated the data and identified eight major clusters. Of note, these eight clusters could be further organized into three larger groups (circled) based on the similarities in gene expression patterns (Figure 1F).

Among these, sub-cluster 5 stood out by being clearly separated from the other clusters in the UMAP plot, suggesting notable differences in its gene expression pattern. Interestingly, this cluster also had the highest proportion of cells originating from PDAC stage III patients, pointing to a possible link with more advanced disease (Figure 1G). We next investigated the stemness markers on this cluster. However, rather than exhibiting classic features of stem-like cells, sub-cluster 5 was characterized by unique gene expression profiles, including genes like TOP2A, PBK, CCNB2, DLGAP5, CENPA, HMMR, MKI67, and BIRC5, all of which are known to be associated with cell proliferation and cancer progression [31,32,33,34,35,36,37,38] (Figure 1H). A further analysis on the distribution of cell cycling and clinical stages across the sub-clusters (Figure 1J) display the characteristics of highly proliferating and less differentiated traits in this sub-cluster. The G2M phase is particularly noteworthy as it is often upregulated in cancer cells to sustain rapid proliferation and overcome cell cycle checkpoints that would otherwise restrict tumor progression [39]. Moreover, GSEA analysis of sub-clusters revealed the dominance of cell cycle-related pathways, including the G2M checkpoint, DNA repair, and E2F targets, emphasizing the proliferative and genomic instability characteristics of sub-cluster 5 (Figure 1I).

### 3.2. Development of a Prognostic Model Capturing the Transitional Phase of PDAC Progression

**Figure 2:**
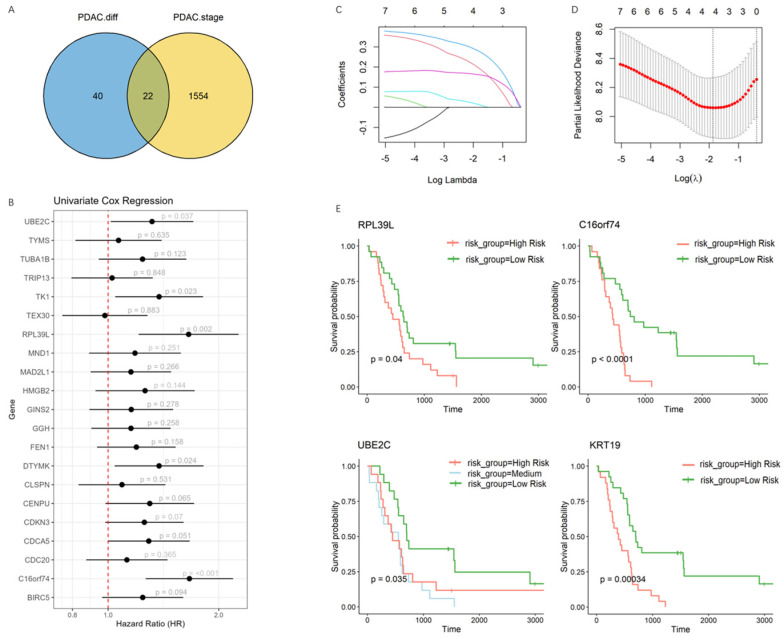
Lasso Cox model construction. (**A**) Overlap of genes associated with cellular differentiation (left circle) and PDAC progression (right circle). (**B**) Identification of prognostic genes in PDAC using univariate Cox regression analysis. (**C**,**D**) Lasso Cox regression analysis of seven input gene lists responsible for PDAC advancement and epithelial identification. (**E**) Kaplan–Meier analysis of patient in stratifying risk groups.

To capture the transitional trait that is reflective of tumor progression, we first selected genes showing continuous upregulation along clinical stages within epithelial cells and discovered 1576 genes which exhibited an enhancement in sub-cluster 5 throughout PDAC progression. Next, we cross-referenced these with genes upregulated in less differentiated cells. The intersection provided a focused list of candidates with potential relevance to both cellular dedifferentiation and PDAC aggressiveness (Figure 2A). We applied subsequent univariate Cox regression analysis to rigorously evaluate prognostic significance (Figure 2B).

To enhance the predictive power of the model and capability of capturing epithelial cell-specific transcriptomic features, we incorporated two well-established epithelial markers, EPCAM and KRT19, both of which are widely used to indicate epithelial status in tumors and are established clinical biomarkers [40,41]. Ultimately, seven genes were incorporated into a Lasso Cox model construction, which allowed us to explore the prognostic significance of these genes (Figure 2C,D).

To that end, four genes were selected to build up the final prognostic model: RPL39L * 0.201 + C16orf74 * 0.283 + UBE2C * 0.0162 + KRT19 * 0.397. Their potential role in clinical prognosis was validated using Kaplan–Meier survival curves to compare survival outcomes between the gene expression level (Figure 2E).

Interestingly, UBE2C, though significant in univariate Cox regression, did not show a statistically significant survival difference between low- and high-risk groups in Kaplan–Meier analysis. This suggests that its prognostic value may not be fully captured by a simple dichotomous risk classification, as UBE2C likely has a more nuanced relationship with survival. Categorizing patients into low-, middle-, and high-risk groups revealed a significant association between UBE2C and survival, indicating that a multi-category approach better reflects its gradual prognostic impact.

### 3.3. Validation and Performance Evaluation of the Prognostic Model in PDAC Progression and Single-Cell Risk Stratification

**Figure 3:**
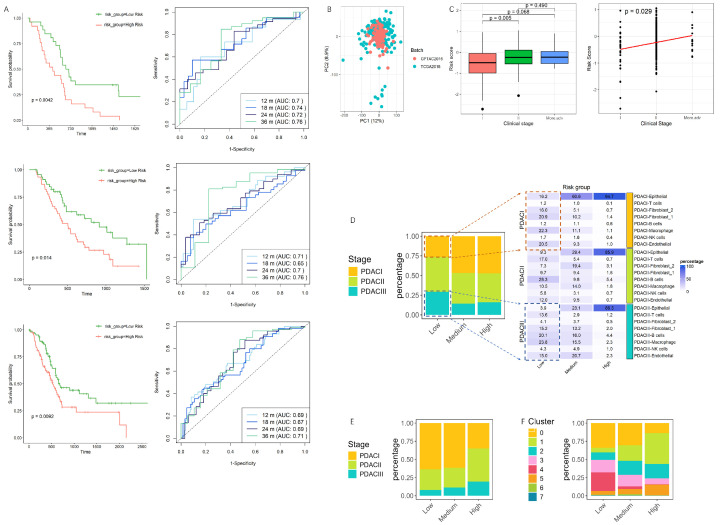
Validation of Lasso Cox model. (**A**) Kaplan–Meier curves and time-dependent ROC analysis for 1- to 3- year survival for patients in high-risk and low-risk group. (**B**) Integration of test cohorts after batch effect removal. (**C**) Risk score progression across clinical stages in PDAC integrated cohort. Risk score distribution across PDAC clinical stages (left), and trend analysis of risk scores (right). (**D**) Distribution of cells across PDAC stages within risk groups. The right plot further breaks down into annotated cell-type composition within each risk group. The value in heat map displays the composition in percentage format. (**E**) Composition of epithelial cells in different PDAC stages in each risk group. (**F**) Composition of epithelial sub-clusters among risk groups.

To validate the predictive power, we cross-validated our model using two clinical datasets, PDAC QCMG and additionally TCGA PanCancer Atlas, specifically focusing on their association with survival outcomes. Risk scores were calculated individually for each patient, and participants were stratified into low-risk and high-risk subgroups accordingly. Across all cohorts, including the training and test sets, patients in the high-risk group exhibited significantly shorter overall survival compared to those in the low-risk group (Figure 3A). Additionally, time-dependent ROC curves provided additional evidence supporting the capability of this model for prognosis prediction in PDAC disease, particularly in distinguishing survival outcomes at early (first 1- to 3-year) time points

Next, we tested the model capability in predicting clinical stage progression. Considering the limited number of PDAC I and PDAC III cases, we combined the two test cohorts after batch effect correction (Figure 3B) and collapsed Phase III and Phase IV into a ‘more advanced’ group. The result exhibited a significant increase in risk scores between PDAC I and PDAC II. However, no statistically significant differences were observed between PDAC I and the more advanced group, nor between Phase II and the more advanced group. Nonetheless, we observed a trend toward higher risk scores in the more advanced group compared to Phase I (Figure 3C, left). Additionally, a Jonckheere–Terpstra test was conducted and showed a significance (*p* < 0.05), indicating a monotonic increase in risk scores as the disease progressed across stages (Figure 3C, right). The observed trend reflects the transitional phase traits which are associated with early to intermediate tumor progression. Its sensitivity to later stages may be inherently limited. It may also be partially attributed to the relatively small sample size within the more advanced group, which is often underrepresented due to clinical and sampling constraints. This could reduce statistical power and the underestimation of true differences in risk scores.

Furthermore, we applied the model on a single-cell level by computing risk scores for individual cells. Cells were categorized into three groups, lower, medium, and high, based on tertiles. Then, we looked at the composition of annotated cell types across PDAC stage within each risk group. As depicted in Figure 3D (right plot), in the low-risk group, PDAC class III had a high percentage of macrophages and B cells, whereas epithelial cells contributed the least (blue squares). Conversely, in earlier-stage PDAC (PDAC I) within the low-risk group, epithelial cells were more abundant (orange squares). As the risk group graded from low to high, the proportion of epithelial cells became more pronounced across all PDAC stages (Figure 3E).

In the risk-grouped epithelial cells, besides sub-cluster 5, two additional clusters—sub-cluster 1 and sub-cluster 2—showed a continuous enrichment as risk scores increased, and sub-cluster 1 exhibited an enhanced epithelial–mesenchymal transition (EMT) signature in previous GSEA analyses (Figure 1I and Figure 3F). These findings confirm that epithelial cells, particularly those with high-risk profiles, play a central role in driving PDAC progression. Importantly, these high-risk epithelial cells appear to be more closely associated with a transitional phase, where they are shifting from a differentiated to a more aggressive, dedifferentiated, and proliferative state.

### 3.4. The Correlation of Marker Genes Expression with PDAC Progress

**Figure 4:**
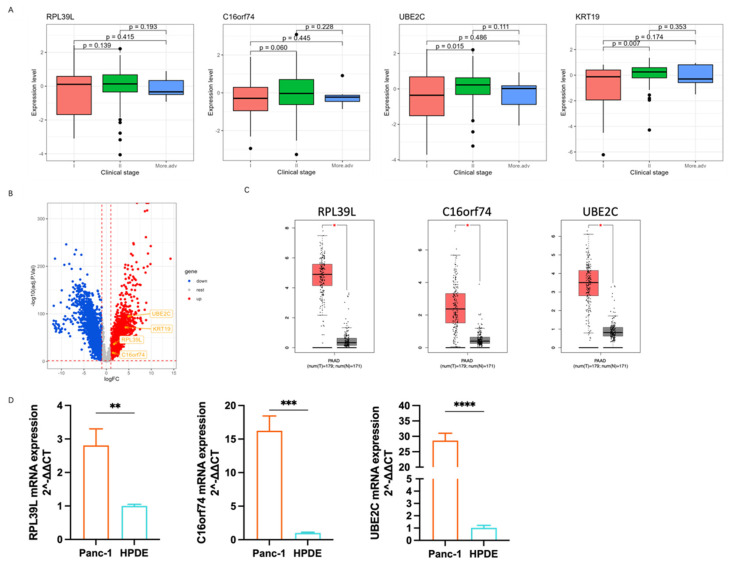
The correlation between the expression of marker genes and PDAC progression. (**A**) The expression of marker genes at different stages of PDAC. (**B**) The differentially expressed genes between PDAC tumor tissue (T) and normal pancreas (N) based on TCGA and GTEx databases. (**C**) The expression levels of RPL39L, C16orf74, and UBE2C in PDAC tumor tissue (T) and normal pancreas (N) based on TCGA and GTEx databases. PAAD = Pancreatic adenocarcinoma. (**D**) The expression levels of RPL39L, C16orf74, and UBE2C mRNA in Panc-1 and HPDE cell lines are shown. Statistical significance is indicated by asterisks: *p* < 0.05 (*), *p* < 0.01 (**), *p* < 0.001 (***), and *p* < 0.0001 (****).

To further investigate the correlation between the expression of marker genes and PDAC progression, we checked their expression across various clinical stages in PDAC patient cohorts from the TCGA database. The results reveal that KRT19, C16orf74, and *UBE2C* were upregulated in PDAC II patients compared to PDAC I, demonstrating the positive correlation between the expression of these marker genes and disease progression (Figure 4A).

The absence of statistical significance in the more advanced group may be attributed to model limitations or the relatively small sample size, as previously noted. Next, we compared their expression with the GTEx database, except *KRT19*, as it is a well-known marker of PDAC, strongly associated with tumor progression and diagnosis. As expected, RPL39L, C16orf74, and UBE2C suggested higher expression levels in the PDAC cohort relative to normal pancreatic tissue (Figure 4B,C). Furthermore, we performed a qPCR (quantitative polymerase chain reaction) to validate the marker genes expression in PCa cancer cell lines Panc-1 and normal pancreatic ductal cells HPDE. The qPCR analysis showed that RPL39L, C16orf74, and UBE2C mRNA was significantly increased in Panc-1 cells compared to HPDE cells (Figure 4C), which highlights the important role of these marker genes in tumorigenesis.

### 3.5. Cell–Cell Communication Network Analysis Highlights Key Signaling Interactions and Centrality in Collagen Pathways

**Figure 5:**
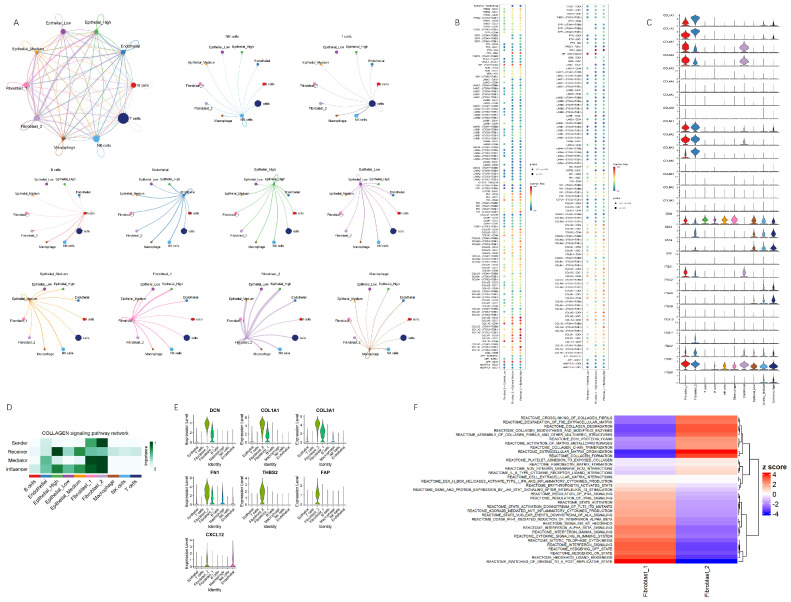
Cell signaling network on fibroblasts and scored epithelial cells. (**A**) Overview of the cell–cell communication network in a weighted, directed format. Edge weights represent interaction strength. The edge weights between different networks display intensity between cell groups. (**B**) Bubble plots of significant interactions from fibroblast_1 (left) and fibroblast_2 (right) to risk-scored epithelial cells. The names on the left represent the molecules of ligand–receptor pairs, with the following color intensity: red indicates higher probability, while green indicates lower probability. (**C**) Gene expression distribution of collagen signaling genes related to L-R pairs. (**D**) Centrality scores of heatmap on collagen signaling. A higher intensity of green suggests greater importance in the network, reflecting dominant roles in sending, receiving, mediating, and influencing intercellular communication. (**E**) Violin plot of genes expression associated with ECM components and the TME. (**F**) Single cell gene set enrichment analysis on collagen and immune-related pathways.

To have a deeper understanding of the mechanisms underlying the behavior of epithelial cells stratified by risk score, we utilized CellChat to examine cell–cell communication dynamics. The results showed that fibroblast_2 exhibited increasingly enhanced signaling interaction with epithelial cells as the risk score escalated (Figure 5A). Notably, this interaction was particularly enriched in high-risk epithelial cells, which are more closely associated with the transitional phase of PDAC. The netVisual_bubble plots revealed that collagen signaling was particularly dominant in the interactions between fibroblasts and epithelial cells, with a marked intensification of signaling between fibroblast_2 and epithelial cells as the risk score increased (Figure 5B), and centrality score analysis reinforced the enhanced communication (Figure 5D). It implies that collagen signaling may actively drive the progression of epithelial cells toward a transitional phase, thereby promoting tumor progression and therapy resistance.

Analyzing those two cell types, we observed that the expression of SDC4, SDC1, ITGB1, ITGA2, and ITGA3 molecules increased as their epithelial cells’ risk score rose, while CD44 expression decreased with higher risk scores (Figure 5C). As for fibroblast_2, we found high expression of extracellular matrix (ECM)-related genes and components of the tumor microenvironment (TME) (Figure 5E), stressing the idea that fibroblast_2-derived collagen is a key mediator of epithelial cell transitions toward a more aggressive, invasive state [42,43]. Pathway analysis further revealed upregulated collagen and ECM pathways in fibroblast_2, whereas fibroblast_1 displayed stronger immune-related signaling, including enriched JAK-STAT, interleukin, interferon (IFN) pathways (Figure 5F). These finding suggest that fibroblast_1 and fibroblast_2 may represent distinct fibroblast subtypes as the UMAP plots suggest, potentially aligning with inflammatory CAFs (iCAFs) and myo-fibroblastic CAFs (myo-like CAFs) in PDAC progression.

### 3.6. Association of the Risk Model with Clinical Outcomes, Tumor Invasiveness, and Drug Response

**Figure 6:**
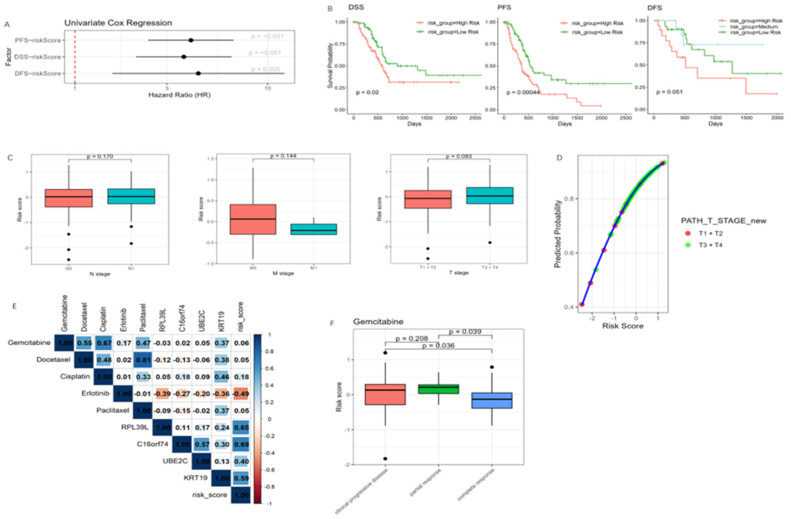
Association of risk model with survival outcomes, TNM staging, and drug response in TCGA. (**A**) Univariate Cox regression analysis of risk score on PFS, DFS, DSS. (**B**) Kaplan–Meier curve for PFS, DFS, and DSS based on risk score. Analysis of DFS conducted a multi-category approach considering the dichotomous may not fully representative. (**C**) Boxplot showing the association between risk score and M-stage, N-stage, and T-stage (from left to right). (**D**) Scatter plot of predicted probability for T-stage based on risk score. Each point represents an individual sample, color-coded by T-stage category (red for T1 + T2 and green for T3 + T4). The LOESS regression curve (blue) highlights a positive trend. (**E**) Correlation between risk scores and Erlotinib sensitivity in pancreatic cancer cell lines. Colored squares indicate significant correlations (*p* < 0.05), with numbers representing the correlation coefficients. (**F**) Association between risk scores and gemcitabine treatment response in PDAC patients.

The univariate Cox regression analysis of the TCGA Pan Atlas cohort demonstrated that the risk model significantly impacts progression-free survival (PFS), disease-free survival (DFS), and disease-specific survival (DSS), with higher risk scores correlating with worse outcomes (Figure 6A). These findings were further supported by Kaplan–Meier survival curves, which showed that high-risk epithelial tumors exhibit a more invasive phenotype, accelerating disease progression and increasing disease-specific mortality (Figure 6B).

Next, we evaluated the model’s ability to predict tumor (T), node (N), and metastasis (M) stages. While risk scores were independent of N- and M-stages, the small number of M1 cases (4 vs. 79 M0) may have limited statistical power. For T-stage, we collapsed the data into two broader groups: ‘T1 + T2’ (smaller and less invasive tumors) and ‘T3 + T4’ (larger and more invasive tumors) groups due to the limited number of T1 and T4 cases. Although no significant association was found, patients in the ‘T3 + T4’ group tended to have higher risk scores (Figure 6C). A scatter plot of T-stage probability against risk scores showed a clear trend that as the risk score rises, the likelihood of a tumor being classified as more invasive increases (Figure 6D).

Moreover, we observed a significant negative correlation between risk scores and the IC50 of Erlotinib in pancreatic cancer cell lines, suggesting that patients with high-risk scores may exhibit increased sensitivity to this drug (Figure 6E). However, in the TCGA dataset, only one patient had received Erlotinib, limiting further analysis. The majority of patients were treated with Gemcitabine, and notably, the complete response group exhibited significantly lower risk scores compared to the partial response and clinical progressive disease groups (Figure 6F).

### 3.7. Immune Infiltration Patterns and Their Association with Risk Scores in PDAC

**Figure 7:**
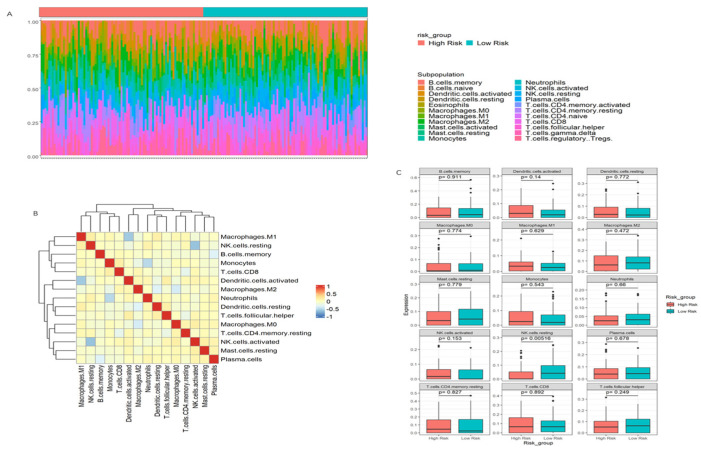
Immune landscape differences between risk groups reveal impaired NK cell activation in high-risk PDAC. (**A**) Overview on immune cell composition grouping by risk score. (**B**) Correlation of immune cell populations in the PDAC tumor microenvironment. (**C**) Comparison of resting and activated NK cell composition across risk groups.

To assess the immune infiltration degree by risk group, we performed CIBERSORT on the TCGA Pan Atlas cohort, which contains 179 cases of transcriptomic data (Figure 7A). The resulting heatmap suggested a negative correlation between several immune cell populations: Macrophages.M1 and activated dendritic cells, resting NK cells and activated NK cells, and monocytes and neutrophils (Figure 7B). This suggests that specific immune cell types may be inversely regulated in the TME of PDAC, with potential implications for tumor progression and immune evasion.

We further examined immune cell composition across the low- and high-risk groups. A striking observation was the significant increase in the number of resting NK cells in the high-risk group compared to the low-risk group (Figure 7C). This finding is particularly relevant, as NK cells play a crucial role in immune surveillance, and their infiltration is often correlated with tumor progression [44]. Interestingly, despite the inverse relationship between resting NK cells and activated NK cells in the TME, we did not observe any significant difference in the composition of activated NK cells between the risk groups, indicating a redistribution of NK cell subsets within the TME, characterized by an increased proportion of resting NK cells in the high-risk group. However, in the absence of activation-specific surface markers and functional transcriptomic profiles, the activation state of NK cells cannot be conclusively characterized.

## 4. Discussion

The construction of a four-gene prognostic model based on single-cell and bulk transcriptomes from PDAC provides a valuable tool for predicting patient outcomes and guiding clinical management, particularly in early or intermediate stages of the disease (before PDAC III). Our model effectively captures tumor progression from early to intermediate phases, aligning with biological and clinical observations. However, its sensitivity to distinguishing later-stage disease appears to be limited, which may be attributed to both biological and statistical factors.

Biologically, our findings suggest that the most significant shift in tumor behavior occurs between Phase I and Phase II, characterized by epithelial plasticity, dedifferentiation, and increased invasiveness [45,46]. This transition aligns with the upregulation of genes linked to the transitional phase of epithelial cancer cells [47,48]. However, as tumors advance into later stages (Phase III), additional mechanisms, such as immune evasion and metastasis, may predominate the tumor behavior [49]. Since our model is built on epithelial-centered transitional genes, its sensitivity to later-stage differentiation may be reduced, as these genes become less relevant in highly aggressive, more heterogeneous tumor states [50].

Statistically, sample imbalance may have contributed to limited sensitivity in later stages, as there was a disproportionately small number of advanced-stage cases, making it difficult to detect significant differences, even if a trend existed. Furthermore, higher variance in the advanced-stage group may have diluted clear associations, impacting statistical significance. While this study included TCGA-based analyses and validation in a representative PDAC cell line, further studies using additional PDAC models and patient-derived samples will be needed to better define the stage-specific relevance of these markers.

Despite these limitations, the significant increase in risk scores from Phase I to Phase II supports the robustness of our model in identifying early transitions to a more aggressive epithelial state.

An essential finding of our study is the shift in receptor usage in epithelial cells as the risk score increases. Specifically, we observed that CD44 expression decreased while SDC1, SDC4, ITGB1, ITGA2, and ITGA3 were upregulated in high-risk tumors. These molecules play crucial roles in cell adhesion, ECM interactions, and tumor invasion. Their high expression levels have been associated with poor differentiation, advanced-stage disease, and increased metastatic potential in PDAC [51,52,53]. The integration of our risk model with clinical progression markers such as PFS, DSS, DFS, and T-stage further supports its strong clinical relevance and association with tumor aggressiveness. Recent studies using co-culture systems have shown that CAFs enhance collagen secretion and establish direct cell–cell interactions that support PDAC cell survival and invasion [54,55]. In our study, we further highlight the critical role of fibroblast–epithelial interactions in PDAC progression by identifying a strong connection between myo-like fibroblasts and high-risk epithelial cells through collagen signaling. This reinforces the importance of fibroblast activity in PDAC pathogenesis, reinforcing the need for fibroblast subtype-specific therapeutic strategies.

We also found a strong correlation between risk scores and drug response. This suggests that the risk model could serve as a predictive biomarker for chemotherapy sensitivity, particularly in distinguishing patients who are under the transitional phase of disease. However, the limited clinical data restricted additional validation. In particular, the limited availability of cases in certain clinical groups, such as patients with advanced-stage disease or those treated with Erlotinib, may influence the reliability and applicability of these findings. Future studies should validate these results in larger, independent cohorts and explore the underlying molecular mechanisms driving these associations.

The immune microenvironment plays a crucial role in PDAC progression, with accumulating evidence indicating a profound immunosuppressive landscape characterized by impaired immune cell function [20,56]. Our study suggests that tumor progression may be associated with an altered NK cell phenotype, potentially leading to impaired immune surveillance during the epithelial transition phase. This aligns with previous reports that PDAC tumors actively suppress NK cell activation through various immunosuppressive mechanisms, including TGF-β signaling and inhibitory receptor expression [57].

The observed increase in resting NK cells in the high-risk group, without a corresponding change in activated NK cells, reflects altered NK cell dynamics within the TME. There may be several explanations for this phenomenon. For example, regulatory T cells and myeloid-derived suppressor cells in the tumor immune microenvironment can inhibit NK cell activation despite increased infiltration by releasing transforming growth factor-beta, interleukin-10, and nitric oxide [58]. Tumor cells can also activate the signal transducer and activator of transcription 3 (STAT3) signaling pathway to suppress NK cell activity [59]. These immunosuppressive processes may be part of a broader immune-evasive strategy adopted by aggressive epithelial cells, contributing to the selective enrichment of resting NK cells. In addition, the increase in resting NK cells in the tumor microenvironment may represent a compensatory response yet potentially exhibits tumor-promoting activity [60]. Due to the limitation of our study, future studies integrating NK cell phenotyping with transcriptomic analysis will be essential to elucidate the underlying mechanisms of this shift, as well as to better define the role of NK cells in immune modulation and risk stratification in this context.

In conclusion, our findings emphasize the importance of integrating molecular profiling with immune landscape analysis to improve risk stratification in PDAC. By identifying key drivers of tumor progression, our study lays the foundation for future research aimed at developing targeted therapies that disrupt fibroblast-mediated tumor interactions and enhance immune responses. Further exploration of fibroblast subtypes and NK cell dysfunction in PDAC may open new ways for improving patient outcomes.

## 5. Conclusions

In conclusion, this study reveals the transcriptomic heterogeneity between subgroups of transitional-phase epithelial cells in PDAC and establishes a gene-based risk model associated with patient survival and disease stage. Furthermore, the TME, including epithelial–fibroblast interactions and immune infiltration pattern, was analyzed in the context of the risk model for its potential role in PDAC progression. Overall, these findings present a promising diagnostic model and highlight potential therapeutic targets for PDAC.

## Data Availability

The datasets used and/or analyzed during the current study are available from the TCGA, GEO, GTEx, and TISCH public databases.

## Abbreviations

The following abbreviations are used in this manuscript:

PDAC	Pancreatic ductal adenocarcinoma
PAAD	Pancreatic adenocarcinoma
PCa	Pancreatic cancer
ECM	Extracellular matrix
TME	Tumor microenvironment
HPDE	Human pancreatic duct epithelial
qPCR	quantitative polymerase chain reaction
NK cells	Natural killer cells
Adjusted *p*-value	*p.adj*
